# Abstract not submitted for online publication

**DOI:** 10.1186/1756-8935-6-S1-P39

**Published:** 2013-03-18

## Abstract not submitted for online publication

